# *HER2* low expression breast cancer subtyping and their correlation with prognosis and immune landscape based on the histone modification related genes

**DOI:** 10.1038/s41598-023-49010-7

**Published:** 2023-12-08

**Authors:** Jia Li, Jingchun Yao, Liqiang Qi

**Affiliations:** 1 Department of Breast Surgical Oncology, Shanxi Province Cancer Hospital/Shanxi Hospital Affiliated to Cancer Hospital, Chinese Academy of Medical Sciences/Cancer Hospital Affiliated to Shanxi Medical University, Xinghualing District, Taiyuan, 030013 Shanxi Province People’s Republic of China; 2 Department of Head and Neck, Shanxi Province Cancer Hospital/Shanxi Hospital Affiliated to Cancer Hospital, Chinese Academy of Medical Sciences/Cancer Hospital Affiliated to Shanxi Medical University, Xinghualing District, Taiyuan, 030013 Shanxi Province People’s Republic of China; 3 Department of Breast Surgical Oncology, Cancer Institute and Cancer Hospital, Chinese Academy of Medical Sciences and Peking Union Medical College, No.17 Panjiayuan, Huawei South Road, Chaoyang District, Beijing, 100021 People’s Republic of China

**Keywords:** Biochemistry, Biological techniques, Biophysics, Biotechnology, Cancer, Cell biology, Chemical biology, Computational biology and bioinformatics, Drug discovery, Genetics, Immunology, Molecular biology, Biogeochemistry, Biomarkers, Diseases, Health care, Medical research, Molecular medicine, Oncology, Pathogenesis, Risk factors, Signs and symptoms

## Abstract

Human epidermal growth factor receptor 2 (*HER2*) plays an important role in diagnosis and treatment of breast cancer (BRCA). The histone modification has been found to be related to the progression of cancer. This study aimed to probe the low *HER2* expression BRCA heterogeneity by histone modification genes. The BRCA data and cell lines were collected from The Cancer Genome Atlas database. Weighted gene co-expression network analysis and non-negative matrix factorization clustering were jointly applied to obtain BRCA clusters. The expression of hub histone modification gene was detected using western blot assay. The gene ontology term and Kyoto Encyclopedia of Genes and Genomes (KEGG) pathway enrichment analysis were performed to reveal functional information. The overall survival analysis was performed using survival and survminer packages, and the immune landscape was mainly analyzed using CIBERSORT software. Totally 43 histone modification genes correlated with survival of BRCA patients with *HER2* low expression were screened. Based on these 43 histone modification genes, the BRCA samples were classified into cluster1, cluster2 and cluster3. Histone modification gene *NFKBIZ* exhibited high expression, while *RAD51* demonstrated low expression in low *HER2* expression BRCA cell. Cluster1 exhibited the best prognosis, while cluster3 had the worse outcomes. Tumor mutational burden (TMB) was remarkably increased in cluster3 group compared to cluster1 and cluster2. Moreover, the relative proportion of 16 immune cell infiltration and 8 immune checkpoint expression were remarkably differential among cluster1, cluster2 and cluster3, and the drug sensitivity exhibited difference among cluster1, cluster2 and cluster3 in BRCA patients with low *HER2* expression. This study identified three *HER2* low expression BRCA clusters with different characteristics based on histone modification genes. The TMB, immune cell infiltration, immune checkpoints and drug sensitivity were different among the three clusters.

## Introduction

Breast cancer (BRCA) is the most frequent type of malignancy among women worldwide, comprising 31% of all female cancers around the globe, and it has the highest mortality rate among all tumors^1^. It has been estimated that approximately 300,590 new cases of BRCA were diagnosed, and about 43,700 deaths occurred in the United States according to the cancer statistics in 2023^2^. Moreover, it has been indicated that BRCA displays intra- and inter-patient tumor heterogeneity^3^. The estrogen receptor (ER), progesterone receptor (PR) and human epidermal growth factor receptor 2 (*HER2*) expression are the routine assessment of BRCA^4^.

*HER2* is a transmembrane receptor tyrosine kinase that belongs to the epidermal growth factor receptor family**.** Approximately 20% of BRCA patients are *HER2*-positive. *HER2* positivity is *HER2* overexpression in BRCA, and it is correlated with tumor aggressiveness and poor prognosis^5^. Anti-HER2 targeted therapies could improve the prognosis of *HER2*-positive BRCA patients. For instance, single-agent trastuzumab (a monoclonal antibody that targets *HER2*) can effectively reduce the progression of *HER2*-positive primary and metastatic BRCA^6^. Moreover, anti-*HER2* reagents have become the standard of treatment for BRCA patients with early or advanced *HER2*-positive^7,8^. *HER2*-low cancers account for about 45 ~ 55% of BRCA^9^. Trastuzumab blockade of the *HER2* pathway in patients with low expression of *HER2* has limited clinical value^10^. Nonetheless, the new anti-*HER2* agents have potential predictive value in *HER2*-low BRCA cell lines^11^, which prompts further development in this setting. In addition, there is biological heterogeneity in BRCA patients with low *HER2* expression. Little is currently known about the biological behavior of BRCA with *HER2* low expression.

Histone modification is the process by which histones undergo methylation, acetylation, phosphorylation and other modifications under the action of related enzymes^12^. Several different classes of histone modifications have been found, such as acetylation, methylation, ubiquitination, carbonylation, adpribosylation, phosphorylation, adenylation, glycosylation and so on^13^. Histone modification can influence the transcription activity of genes. Histone modifications play important roles in many cellular processes, such as cell cycle, embryonic development, chromatin structure, differentiation and chromosome stability^14^. Furthermore, previous studies have revealed that dysregulation of histone modification is critically involved in the progression of several diseases, especially in cancer^15^.

Thus, in this work, we aimed to elucidate the heterogeneity of low *HER2* expressing BRCA at a molecular level based on the aberrantly expressed histone modification associated genes via the methods of bioinformatics research. Our study could provide valuable a theoretical basis for diagnosis and treatment of BRCA.

## Materials and methods

### Data sources

The mRNA expression profiling data of 420 BRCA patients with low *HER2* expression (BRCA patients with *HER2* expression of 1 + and 2 +), along with the corresponding clinical information, were downloaded from The Cancer Genome Atlas (TCGA, https://tcga-data.nci.nih.gov/tcga/) database (Table S1). The maf files of BRCA were also downloaded for subsequent analysis.

### Weighted gene co-expression network analysis (WGCNA)

According to the expression values of genes, the top 25% of genes were screened by variance analysis to the WGCNA using “WGCNA” function package^16^ in R language (version 4.1.0, the same below). Pearson correlation coefficients were calculated for each gene, and selected appropriate soft threshold β. One-step method was applied to build a gene network, and the adjacency matrix was transformed into a topological overlap matrix (TOM), and hierarchical clustering was applied to produce a hierarchical clustering tree. The significance between genes and clinical information was measured by calculating the significance between the genes and nodules, and gene modules were obtained via calculating the significant association between modules and traits.

### Functional enrichment analysis

The gene ontology (GO, including Biological Process (BP), Molecular Function (MF), Cellular Component (CC)) term and Kyoto Encyclopedia of Genes and Genomes (KEGG) pathway enrichment analysis were employed to analyze the function of hub genes associated with cluster1, cluster2 and cluster3 using the “clusterProfiler” function package in R language^17^. The significantly enriched pathways were screened by *p* < 0.05.

### Survival analysis

The R language survival package and survminer package were used to estimate the overall survival of patients based on the Kaplan–Meier method. The significance of differences in survival among different groups was tested by a log-rank test.

### Immune cell infiltration

The software CIBERSORT^18^ was employed to calculate the relative proportions of 22 immune cells in the samples. CIBERSORT software characterizes the composition of immune infiltrating cells according to gene expression matrices using a deconvolution algorithm based on a preset set of 547 barcode genes. The sum of all estimated immune cell type proportions in each sample equals one.

### Differential gene analysis

The BRCA cell lines with high *HER2* expression (*HER2* +) (md453, SKBR3, BT474) and low *HER2* expression (*HER2*-) (mcf7, MDA_MB_468, MDA_MB_231) were obtained from the cell database. The differential gene analysis between *HER2*( +) and *HER2*(-) BRCA cells was performed using “limma” package of R language (version 4.2.0). The differentially expressed genes (DEGs) were screened using the |log_2_FC|> 1 and *p* < 0.05.

### Cell lines and cell culture

The human BRCA cell lines MDA-MB-231 (*HER2*-) and SKBR3 (*HER2* +) were purchased from Procell Life Science & Technology Co.,Ltd. (Wuhan, China). MDA-MB-231 was cultured in Dulbecco's modified eagle medium (DMEM, PM150210, Procell), containing 10% fetal bovine serum (FBS, 164,210, Procell) and 1% penicillin/streptomycin (P/S). MDA-MB-231 was maintained in McCoy’s 5A medium (PM150710, Procell), supplemented with 10% FBS and 1% P/S. All cells were maintained at 37 °C in 5% CO_2_ incubator.

### Western blot

Proteins were extracted from cells using a homogenizer in radio immunoprecipitation assay (RIPA) buffer (R002, Solarbio), supplemented with protease inhibitors. The western blot was consistent with previous methods^19^. The first antibodies were Her2 (ab134182, 1:10,000, abcam), GAPDH (UM4002, 1:2000, Youkang), NFKBIZ (53,174, 1:500, SAB) and RAD51 (ab133534, 1:10,000, abcam). The second antibodies were Goat Anti-Rabbit IgG-HRP (bs-0295G-HRP, 1:3000, Bioss) and Goat Anti-Mouse IgG-HRP (bs-0296G-HRP, 1:3000, Bioss). The internal reference was GAPDH. Protein bands were detected in multi-purpose chemiluminometer (Chemi6000, Clinx Science Instruments Co., Ltd. Shanghai, China), and the gray values of the bands were analyzed using image J software.

### Statistical analysis

All statistical analyses were performed using R software (Version 4.2.0). The difference among various groups was determined using t.test. The result was considered statistically significant when the *p* < 0.05.

## Results

### Based on histone modification genes, identification of low HER2 expression BRCA clusters

**Figure 1:**
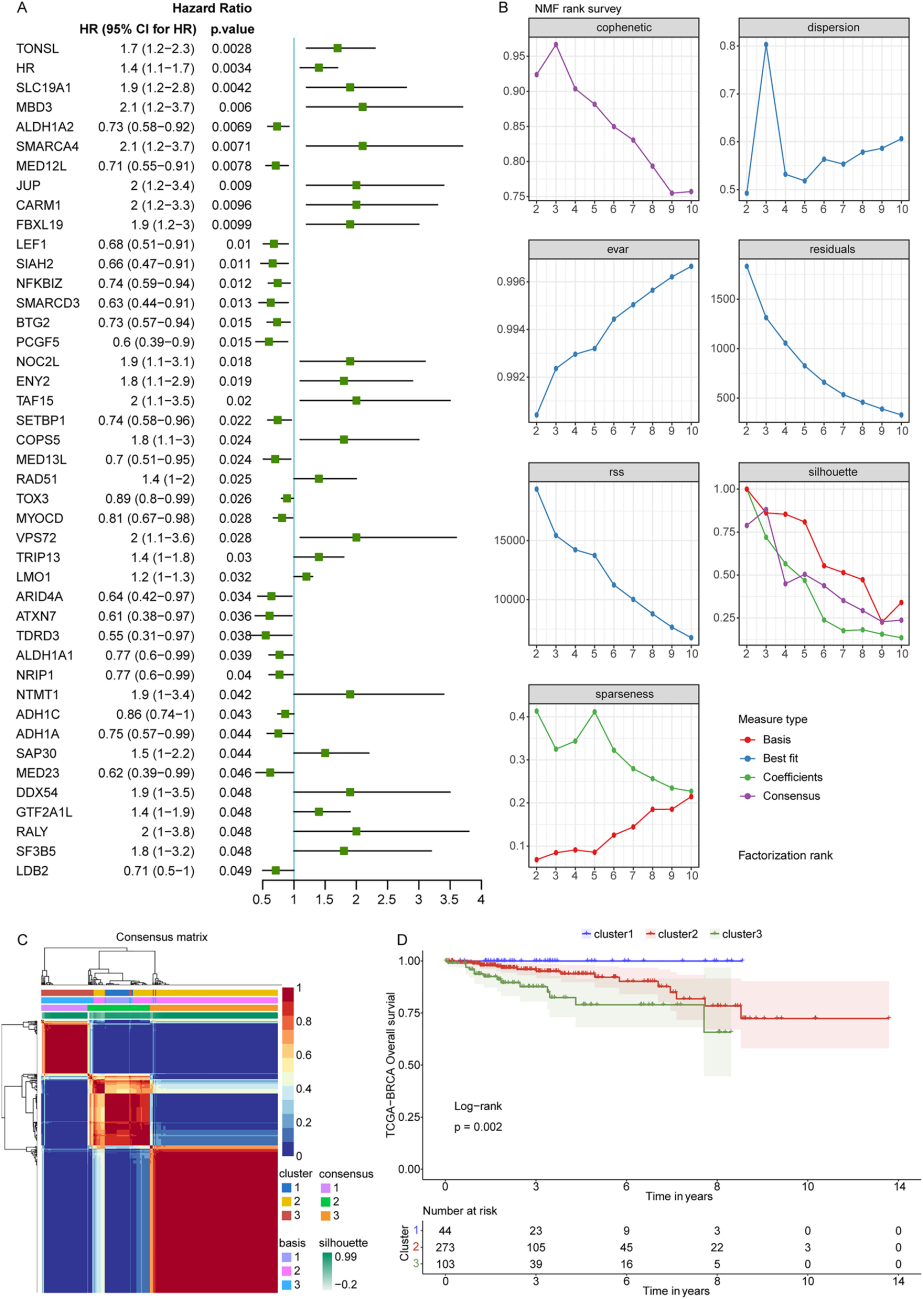
Screening of histone modification genes associated with BRCA patients with low *HER2* expression and identification of low *HER2* expression BRCA clusters. (**A**) Forest plot of univariate Cox regression. (**B**) The non negative matrix factorization (NMF) clustering of BRCA patients with low HER2 expression according to 43 43 histone modification genes. (**C**) The heatmap of NMF cluster classification. (**D**) The Kaplan Meier survival curve of BRCA patients with low HER2 expression in cluster1, cluster2 and cluster3.

Firstly, we searched the gene sets with “histone modification” in MSigDB database and downloaded 736 histone modification genes (Table S2). The univariate Cox regression analysis showed that in the TCGA database, 43 genes were significantly correlated with survival of BRCA patients with low *HER2* expression among these 736 histone modification genes (Fig. 1A). Subsequently, we performed non negative matrix factorization (NMF) clustering based on these 43 histone modification genes, the results indicated that the low *HER2* expression BRCA samples were successfully classified into cluster1, cluster2 and cluster3 (Fig. 1B,C). The BRCA patients in cluster1 exhibited the best prognosis, while in cluster3 had the worse outcomes (Fig. 1D).

### The 19 histone modification genes were differentially expressed among cluster1, cluster2 and cluster3

**Figure 2:**
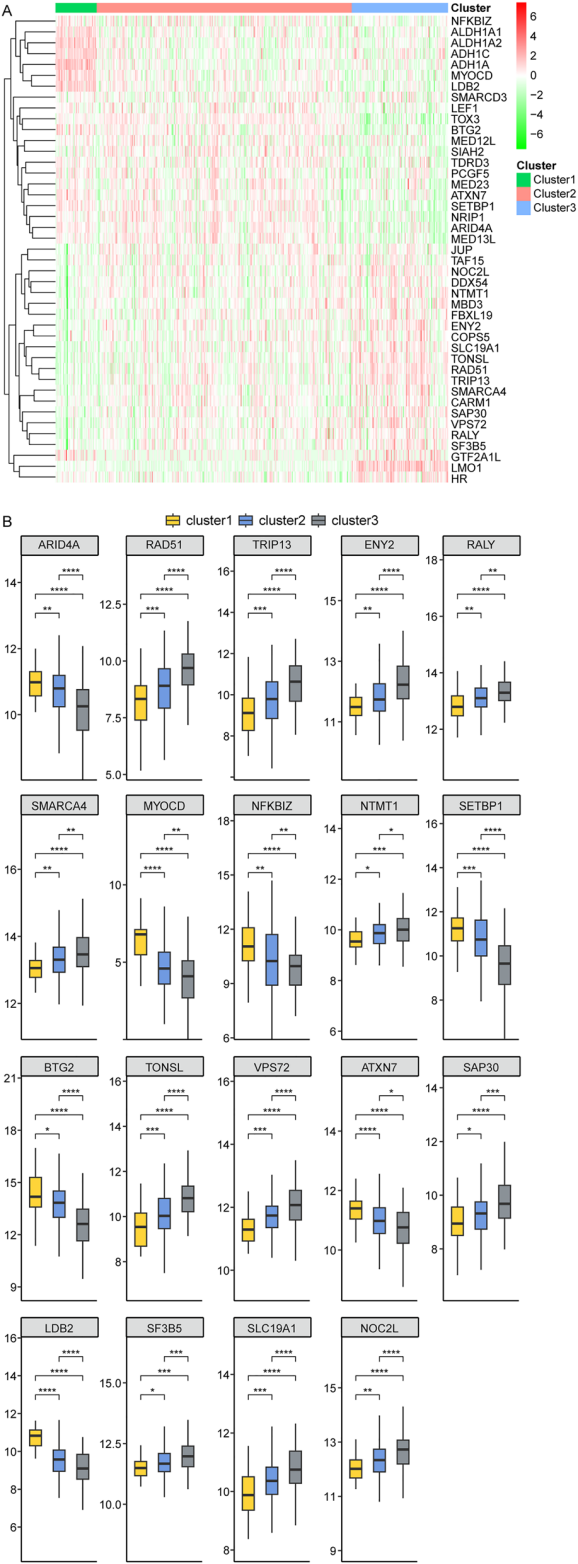
The 19 histone modification genes expression were differential among cluster1, cluster2 and cluster3. (**A**) The heatmap of 43 protein modification gene expression in low HER2 expression BRCA patients in cluster1, cluster2 and cluster3. (**B**) The expression of 19 protein modification gene in low HER2 expression BRCA patients in cluster1, cluster2 and cluster3.

In the TCGA database, we analyzed the 43 histone modification gene expression in cluster1, cluster2 and cluster3 (Fig. 2A), and found that 19 gene expressions were significantly differential among three groups. *AR1D4A*, *MYOCD*, *NFKBIZ, SETBP1*, *BTG2*, *ATXN7*, *LDB2* expressions were highest in cluster1 and lowest in cluster3 (cluster1 > cluster2 > cluster3), while the *RAD51*, *TRIP13*, *ENY2*, *RALY*, *SMARCA4*, *NTMT1*, *TONSL*, *VPS72*, *SAP30*, *SF3B5*, *SLCLUSTER19A1*, *NOCLUSTER2L* expressions were highest in cluster3 and lowest in cluster1 (cluster3 > cluster2 > cluster1) (Fig. 2B).

**Figure 3:**
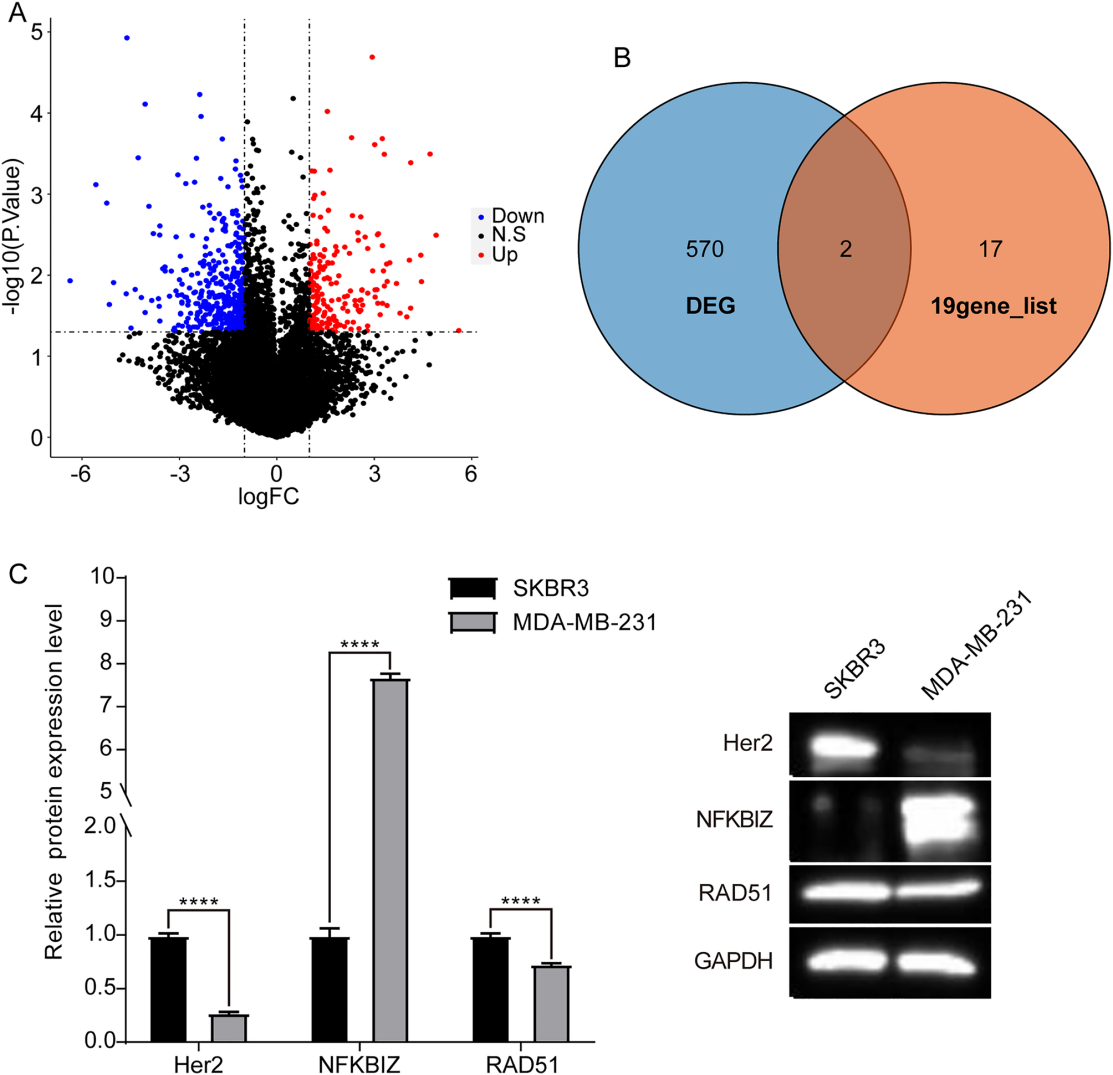
The expression of histone modification genes HER2( +) and HER2(-) BRCA cell lines. (**A**) Differentially expressed genes (DEGs) between HER2( +) and HER2(-) BRCA cell lines. (**B**) Overlapping genes between DEGs and 19 19 histone modification gene groups. (**C**) The expression of Her2, *NFKBIZ* and *RAD51* in BRCA cell lines SKBR3 (HER2 +) and MDA-MB-231 (HER2-).

Next, we collected *HER2*( +) (md453, SKBR3, BT474) and *HER2*(-) BRCA cell lines (mcf7, MDA_MB_468, MDA_MB_231), and identified totally 572 DEGs between *HER2*( +) and *HER2*(-) BRCA cell lines (Fig. 3A, Table S3). A cross-over analysis showed that *NKFBIZ* and *RAD5O* were overlapping genes between 572 DEGs and 19 histone modification gene groups (Fig. 3B), indicating that the expression of *NKFBIZ* and *RAD5O* was significantly differential between *HER2*( +) and *HER2*(-) BRCA cells. To verify this result, we analyzed the *NKFBIZ* and *RAD5O* expression in human BRCA cell lines SKBR3 (*HER2* +) and MDA-MB-231 (*HER2*-). As depicted in Fig. 3C, the expression of *NFKBIZ* protein was significantly higher in MDA-MB-231 cells compared to SKBR3 cells. Conversely, *RAD51* protein expression was lower in MDA-MB-231 cells than in SKBR3 cells. These results suggested that the histone modification gene *NFKBIZ* exhibited high expression, while *RAD51* demonstrated low expression in *HER2*(-) BRCA cells.

### The potential function of hub genes associated with cluster1, cluster2 and cluster3, respectively

**Figure 4:**
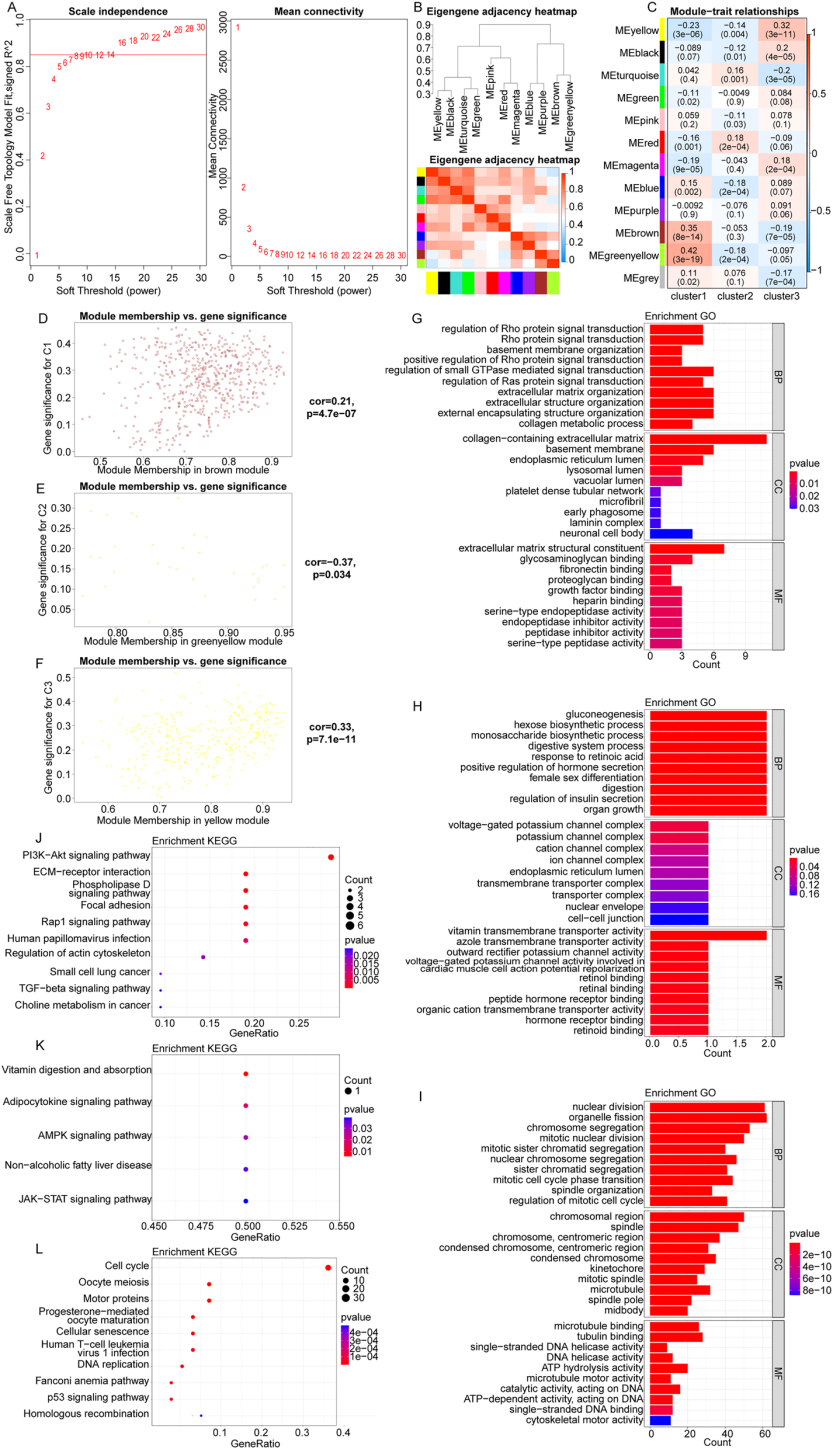
The potential function of hub genes associated with cluster1, cluster2 and cluster3, respectively. (**A**) The soft threshold β of weighted gene co-expression network analysis (WGCNA). (**B**) The clustering result of gene module. The top half is a hierarchical clustering dendrogram of genes, the bottom half is gene modules, and module colors represent the color of each module. (**C**) The correlation of gene modules with cluster1, cluster2 and cluster3. The leftmost color block represents the module. The rightmost color bar represents the correlation range, red represents positive correlation, blue represents negative correlation and darker color represents stronger correlation. (**D**–**F**) The correlation between gene significance and module membership in cluster1, cluster2 and cluster3. (**G**–**I**) The hub genes in cluster1, cluster2 and cluster3 significantly enriched the top 10 GO terms. The horizontal axis indicates the number of genes enriched, and the vertical axis indicates the GO terms. BP: Biological Process; MF: Molecular Function; CC: Cellular Component. (**J**–**L**) The hub genes in cluster1, cluster2 and cluster3 significantly enriched in top 10 KEGG signaling pathways. The horizontal axis indicates the number of genes enriched, and the vertical axis indicates the KEGG pathways.

In the TCGA database, the BRCA patients with low *HER2* expression were subjected to WGCNA, and the soft threshold β was 16 (Fig. 4A). Next, we established a gene network and obtained 11 gene modules (Fig. 4B). The correlation between gene modules and cluster1, cluster2 and cluster3 were presented in Fig. 4C. The high correlation between gene significant and module membership showed that the hub genes in brown, green yellow and yellow modules tend to be highly correlated with cluster1, cluster2 and cluster3, respectively (Fig. 4D–F). Thus, according to module membership > 0.8 and module significance > 0.2, we screened the hub genes associated with cluster1, cluster2 and cluster3 in brown, green yellow and yellow modules, respectively (Table S4).

The enrichment analysis showed that the hub genes associated with cluster1 were significantly enriched in 113 GO terms and 12 KEGG pathways (Table S5). The hub genes associated with cluster2 were remarkably enriched in 369 GO terms and 5 KEGG pathways (Table S6). The hub genes correlated with cluster3 were observably enriched in 493 GO terms and 19 KEGG pathways (Table S7). The hub genes in cluster1, cluster2 and cluster3 observably enriched the top 10 GO terms, and KEGG pathways were displayed in Fig. 4G–L, respectively.

### The cluster1, cluster2 and cluster3 were correlated with tumor mutational burden (TMB) in BRCA patients with low HER2 expression

**Figure 5:**
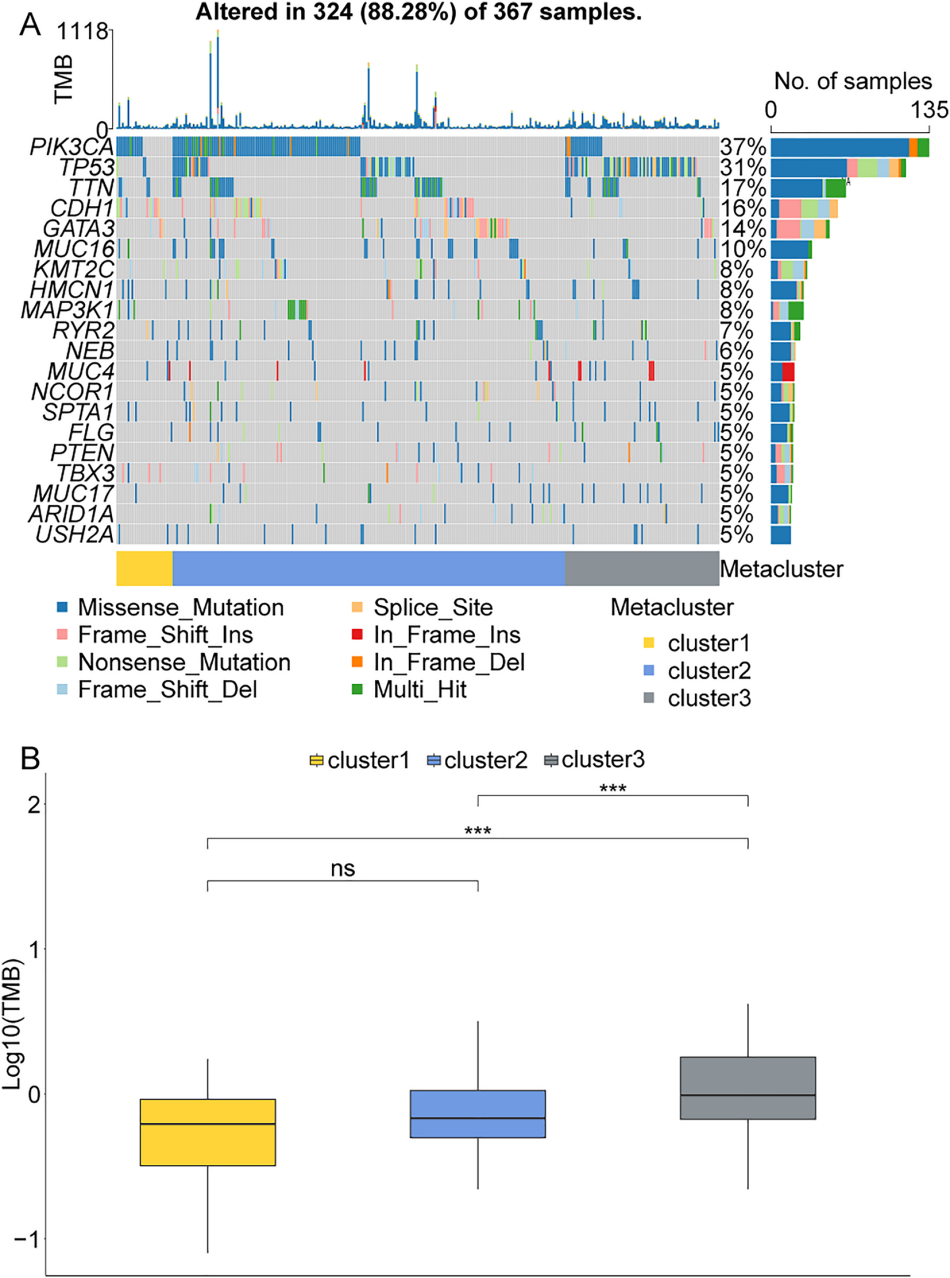
The cluster1, cluster2 and cluster3 were correlated with TMB in BRCA patients with low *HER2* expression. (**A**) The levels of somatic mutations in cluster1, cluster2 and cluster3. (**B**) The levels of tumor mutational burden (TMB) in cluster1, cluster2 and cluster3.

Furthermore, in the TCGA database, we analyzed the somatic mutations and calculated the TMB in cluster1, cluster2 and cluster3. As shown in Fig. 5A, *PIK3CA* and *TP53* had the highest mutation rate in cluster2 and cluster3, respectively. The level of TMB was remarkably increased in cluster3 compared to cluster1 and cluster2 (Fig. 5B).

### The cluster1, cluster2 and cluster3 were correlated with immune cell infiltration and immune checkpoints in BRCA patients with low HER2 expression

**Figure 6:**
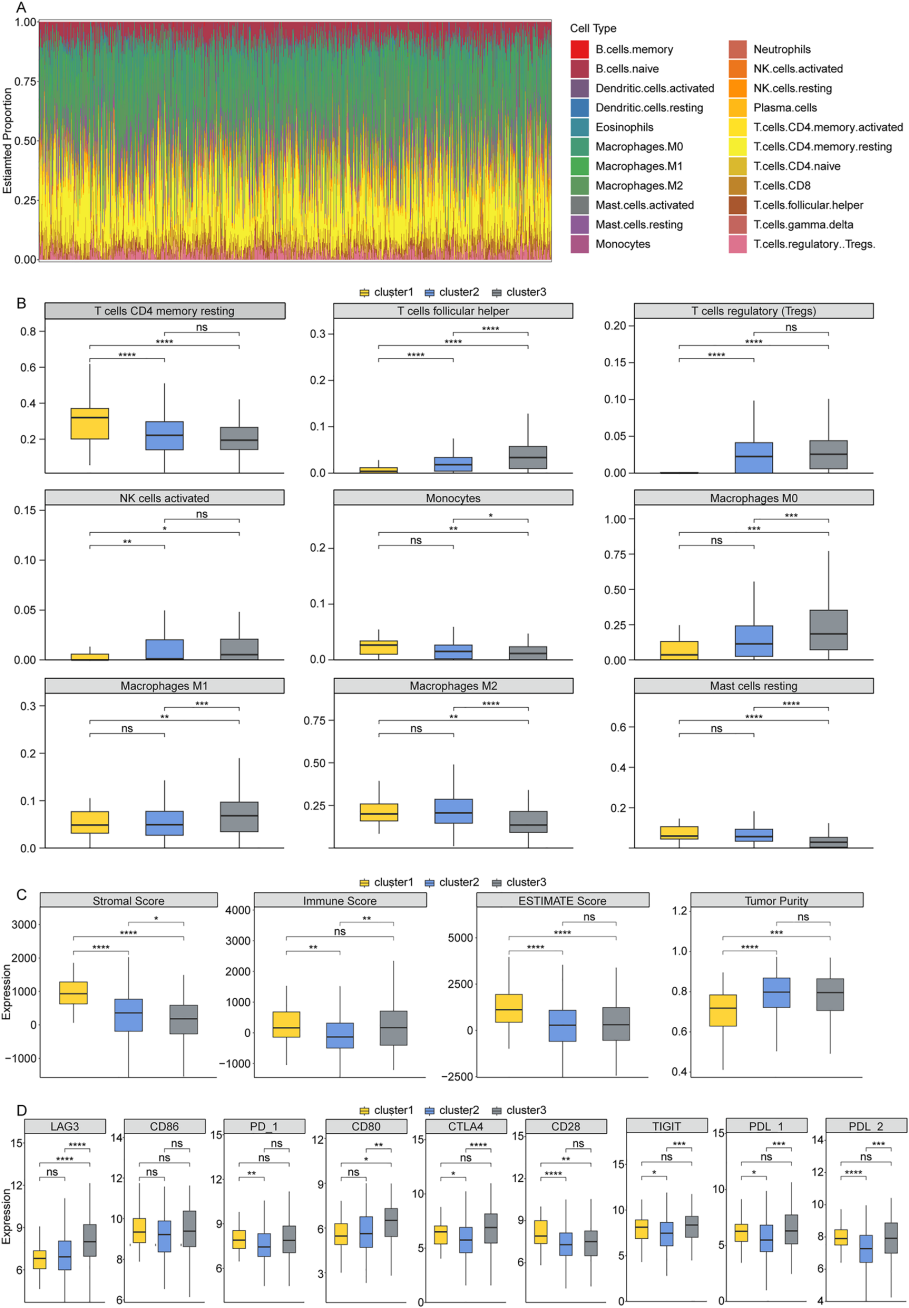
The cluster1, cluster2 and cluster3 were correlated with immune cell infiltration and immune checkpoints in BRCA patients with low *HER2* expression. (**A**) The relative proportion of 22 immune cells in low HER2 expression TCGA-BRCA dataset. (**B**) The relative proportion of 22 immune cells in cluster1, cluster2 and cluster3. (**C**) The levels of ImmuneScore, ESTIMATEScore, StromalScore and TumorPurity in cluster1, cluster2 and cluster3. (**D**) The expression of 9 immune checkpoints (LAG3, CD86, PD-1, CD80, CTLA4, CD28, TIGIT, PDL-1, PDL-2) in cluster1, cluster2 and cluster3.

The relative proportion of 22 immune cells in low *HER2* expression TCGA-BRCA dataset was presented in Fig. 6A. Compared to cluster1 group, the relative proportion of T cells CD4 memory resting was significantly decreased in cluster2 and cluster3 group, and the relative proportions T cells regulatory (Tregs) and NK cells activated were remarkably increased in cluster2 and cluster3 (Fig. 6B). The relative proportion of T cells follicular helper was highest in cluster3 and lowest in cluster1 (cluster3 > cluster2 > cluster1, Fig. 6B). Moreover, compared to cluster1 and cluster2, the relative proportions of Monocytes, Macrophages M2 and Mast cells resting were dramatically reduced in cluster3, and the relative proportions of Macrophages M0 and Macrophages M1 were memorably elevated in cluster3 (Fig. 6B). In addition, the levels of Stromal Score and ESTIMATEScore were significantly increased, and the TumorPurity level was remarkably reduced in cluster1 compared to cluster2 and cluster3 (Fig. 6C). And the Immune score level was elevated in cluster1 and cluster3 compared to cluster2 (Fig. 6C). We also discovered that *LAG3* and *CD80* expressions were significantly increased in cluster3 compared to cluster1 and cluster2 (Fig. 6D). Compared to cluster1, PD-1 expression was reduced in cluster2, and tCD28 expression was decreased in cluster2 and cluster3 (Fig. 6D). Moreover, compared to cluster2, CTLA4, TIGIT, PDL-1 and PDL-2 expressions were dramatically elevated in cluster1 and cluster3 (Fig. 6D).

### The drug sensitivity exhibited different among cluster1, cluster2 and cluster3 in BRCA patients with low HER2 expression

**Figure 7:**
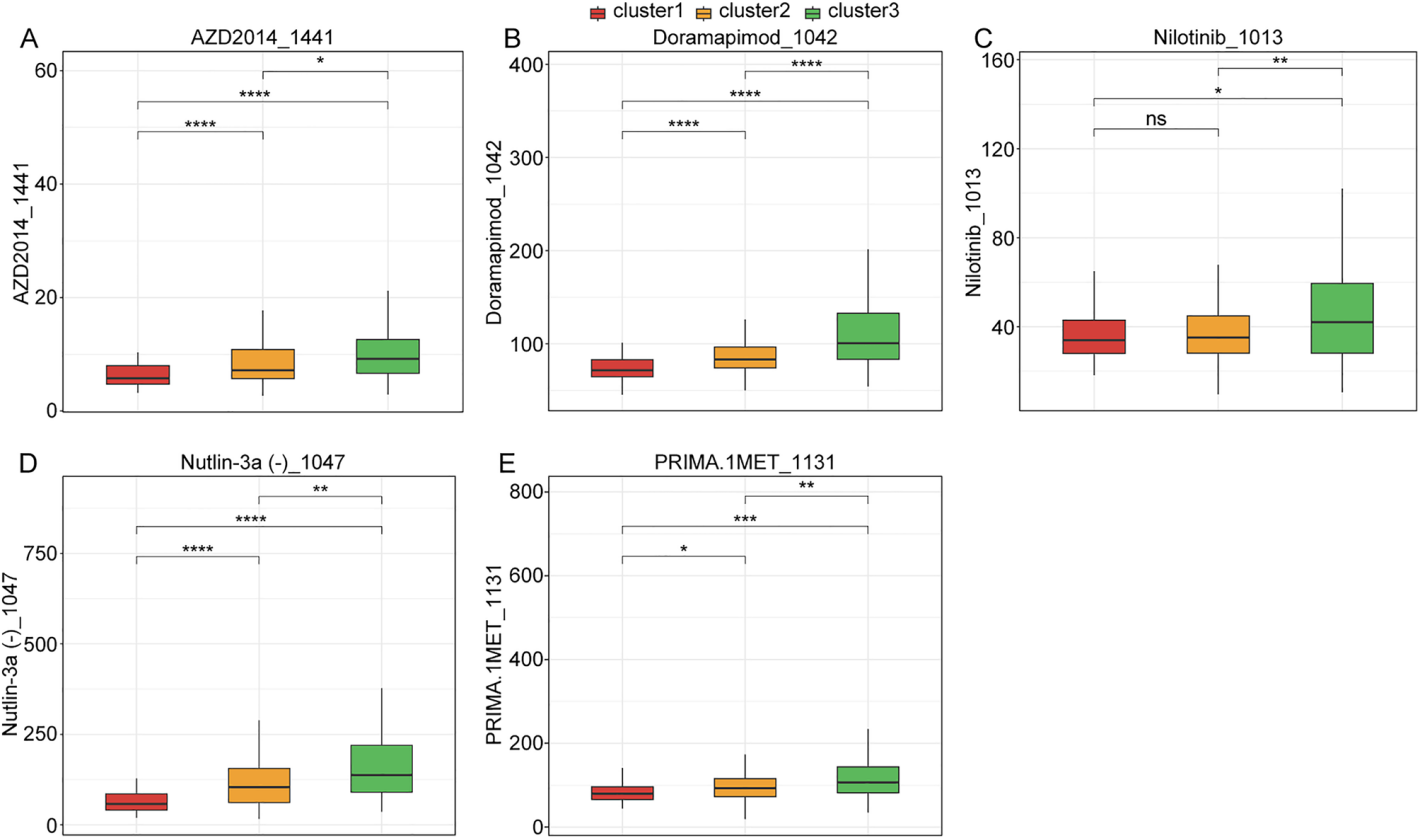
The drug sensitivity exhibited differences among cluster1, cluster2 and cluster3 in BRCA patients with low *HER2* expression. (**A**–**E**) The IC50 of AZD2014_1441, Doramapimod_1042, Nilotinib_1013, PRIMA.1MET_1131, Nutlin-3a (-)_1047 in cluster1, cluster2 and cluster3.

Finally, in the low *HER2* expression TCGA-BRCA dataset, we analyzed the correlation between drug sensitivity and cluster1, cluster2 and cluster3 using “oncoPredict” package in the R language^20^. The “oncoPredict” package included totally 198 drugs. The results showed that the IC50 values of AZD2014_1441, Doramapimod_1042, Nutlin-3a (-)_1047 and PRIMA.1MET_1131 were highest in cluster3 and lowest in cluster1 (cluster3 > cluster2 > cluster1), and the IC50 value of Nilotinib_1013 was significantly increased in cluster3 compared to cluster1 and cluster2 (Fig. 7A–E).

## Discussion

As is well known, BRCA is a diverse disease with different histological tumor subtypes^21^, and the subtypes of *HER2* low expression BRCA have no explicit conceptual framework at molecular levels. In the present study, we identified three *HER2* low expression BRCA clusters according to the histone modification genes and explored their clinical characteristic and immune landscape.

Firstly, based on the 43 histone modification genes that were correlated with survival of BRCA patients with low *HER2* expression, we clustered BRCA patients with low *HER2* expression into three categories (cluster1, cluster2 and cluster3) by NMF clustering analysis. BRCA patients with low *HER2* expression in cluster1 exhibited the best prognosis, while in cluster3 had the worse outcomes. Moreover, we discovered that the 7 histone modification gene expressions were highest in cluster1 and lowest in cluster3, while the 12 histone modification gene expressions were highest in cluster3 and lowest in cluster1 (cluster3 > cluster2 > cluster1). These results suggested that *BRCA* patients with low *HER2* expression could be clustered by histone modification gene, and histone modification gene expressions might preliminarily predict the outcome of BRCA patients with low *HER2* expression patients.

The enrichment analysis showed that the hub genes associated with cluster1 were significantly enriched in signal transduction (Rho protein signal transduction) and immune (PI3K-Akt signaling pathway) signaling pathways. The RHO family belongs to the RAS superfamily of guanine nucleotide-binding proteins. It has been demonstrated that the members of the RHO family were important regulatory molecules that couple changes in the extracellular environment to intracellular signal transduction pathways^22^. Rho GTPases could regulate apoptosis, proliferation, migration, metabolism, tumor microenvironment and cancer cell stemness to promote the initiation and progression of cancers^23^. Noteworthy, the extracellular matrix (ECM) could activate the Rho GTPase signaling upstream of Rho GEFs/GAPs^23^. Yang et al. found that Rho family small GTPases could activate the PI3K through interaction^24^. In BRCA with amplification or mutation of GEF P-REX1, PI3K activation can promote the cancer migration and growth of cancer cells^25^. We also found that the hub genes associated with cluster1 were enriched ECM-receptor interaction and PI3K-AKT signaling pathway. Considering the BRCA patients with low *HER2* expression in cluster1 exhibited the best prognosis compared to cluster2 and cluster3, we hypothesized that the tumor-promoting pathways might be inhibit in BRCA with low *HER2* expression in cluster1.

The cluster2 was remarkably enriched in metabolic related signaling pathway. It has been reported that metabolic alterations with high glycolytic rates were observed in multiple malignancies^26^. In acidic conditions, glycolytic cancer cells can exhibit a non-glycolytic phenotype through an intracellular accumulation of lactic acid^27^. Reversing the glycolytic state to OxPhos can induce cancer cell death^28^. In addition, the limited glucose could promote the death of cancer cells. The cells could switch energy metabolism from mitochondrial respiration to glycolysis when mitochondrial dysfunction or hypoxia occurs, thereby sustaining the growth of tumor^29^. Besides glucose metabolism, fatty acid metabolism has been demonstrated to enhance the lipid synthesis, catabolism and storage, thus promoting the occurrence and progression of tumor^30^. Cancer cells acquired fatty acid from exogenous sources or endogenously synthesize in a dysfunctional manner via the lipogenic pathway, causing excessive accumulation of lipids or increased in saturated and unsaturated acquired fatty levels, thereby disrupting homeostasis and promoting cellular stress^31^. Tang et al. reported that fatty acid metabolism-related genes were closely correlated with the prognosis, immune landscape and immunotherapeutic implications in BRCA^32^. We discovered that the hub genes associated with cluster2 were enriched in metabolic pathways, such as hexose biosynthetic process, glucose metabolic process, monosaccharide metabolic process and lipid transporter activity. These evidences indicated that regulating the metabolic pathways of BRCA might be beneficial to inhibit the progression of BRCA with low *HER2* expression in cluster2.

The cluster3 was closely correlated with cell cycle signaling pathways. In BRCA 4T1 cells, the cells were arrested in the S phase because of the percentage of cells in S phase increased and cells in G1/G0 phase decreased after treating with resveratrol^33^. Daphnoretin could remarkably increase the p21 level and decrease cyclin E and CDK2 levels, and then arrest the cell cycle at the S phase in BRCA^34^. The CDCD20 down-regulation combined with radiation could restrain proliferation, aggravate DNA damage, increase G2/M arrest, and promote apoptosis of HCC cells to a greater extent, and the relative survival fraction of hepatoma cells with p53 mutated Hep3B decreased with increasing irradiation dose^35^. Thus, cell cycle pathways play important roles in the progression of cancers, including BRCA. In this study, we discovered that the hub genes associated with cluster3 were enriched in cell cycle related signaling pathways, such as oocyte meiosis, DNA replication, cellular senescence, p53 signaling pathway and so on. Accordingly, regulating the cell cycle pathways of BRCA might be beneficial to inhibit the progression of BRCA with low *HER2* expression in cluster3.

The somatic mutation analysis showed that *PIK3CA* and *TP53* had the highest mutation rate in cluster2 and cluster3, respectively. It has been known that presence of somatic *PIK3CA* or *TP53* mutations could promote cancer progression in BRCA^36,37^. *PIK3CA* mutations were considered an early event in BRCA development since they have been discovered in tiny tumors and non-invasive precursor lesions^38^. Reinhardt et al. reported that the highest frequencies (> 30%) of *PIK3CA* gene mutations were detected in steroid hormone-receptor (SHR)-positive *HER2*-negative and G1 and G2 BRCA^39^. However, the significant association between presence of PIK3CA mutations and recurrence-free interval events at 5 years was not observed^39^. Thus, further research was required to delve the role of *PIK3CA* gene mutations in prognosis of BRCA patients with low *HER2* expression. *TP53* is a tumor suppressor gene that is frequently altered in breast cancer and other malignancies^37^. In BRCA, *TP53* is the most commonly mutated gene, accounting for roughly 30% of all cases^36^. The cancer patients with *TP53* mutation were more likely to acquire immune escape and had a poor prognosis^40^, which indicated that the BRCA patients in cluster3 exhibited worse prognosis might be related to *TP53* mutation. Moreover, we also discovered that the level of TMB was remarkably increased in cluster3 group compared to cluster1 and cluster2. TMB has been reported to correlate with the objective response rate to immunotherapy (PD-1 inhibitors)^41^. TMB levels have been linked to better clinical benefit with immune checkpoint inhibitors (ICIs) in many malignancies, and TMB and PD-L1 expression are independent predictors of ICIs response with little correlation across multiple tumors^42–44^. However, in BRCA, the patients with high TMB level had poorer prognosis and survival probability than patients with low TMB level^45^, which suggested the in cluster3, the BRCA patients with low *HER2* expression had worse prognosis than patients in cluster1 and cluster2 was reasonable.

Moreover, immune cell infiltration could regulate the progression of tumor and display potential prognostic value in tumor microenvironment^46^. Immune checkpoints were negative regulators in immune system. They participated in the prevented autoimmunity and protected tissues from immune damage^47^. It has been demonstrated that inhibition of immune checkpoints could enhance the anti-tumor immunity in cancers^48,49^. Thus, we next analyzed the immune landscape and immune checkpoint expression in cluster1, cluster2 and cluster3. Immune cell infiltration analysis showed that the proportion of immune cell infiltration exhibited different among cluster1 cluster2 and cluster3 of BRCA patients with low *HER2* expression. Furthermore, the stromal score, ESTIMATE score and tumor purity has also different between cluster1 and cluster3 of BRCA patients with low *HER2* expression. In addition, the expressions of some immune checkpoint were differential among cluster1, cluster2 and cluster3. Collectively, the above pieces of evidence indicated that the immune heterogeneity existed among the cluster1, cluster2 and cluster3. Finally, we also found that the IC50 of some drugs were also differential among cluster1, cluster2 and cluster3, which suggesting stratification of BRCA patients with low *HER2* expression into different subtypes enable clinicians to tailor treatments for individual patients.

Even though we classified BRCA patients with low *HER2* expression into cluster1, cluster2 and cluster3 based on these 43 histone modification genes and analyzed the prognosis, immune cell infiltration and immune checkpoint expressions in patients in cluster1, cluster2 and cluster3, biological research in clinical BRCA samples with high and low HER2 expression were required to validate our key findings.

## Conclusion

In conclusion, we revealed the tumor heterogeneity and identified three clusters in BRCA with low *HER2* expression based on the histone modification genes. Moreover, the TMB, immune cell infiltration, immune checkpoints and drug sensitivity were different among the three clusters. Our study provided a new classification system of BRCA with low *HER2* expression, which helps improve the prognosis and promote clinical management.

### Supplementary Information

Supplementary Table S1.

Supplementary Table S2.

Supplementary Table S3.

Supplementary Table S4.

Supplementary Table S5.

Supplementary Table S6.

Supplementary Table S7.

Supplementary Figures.

## Data Availability

The data that support the findings of this study are available in The Cancer Genome Atlas (TCGA, https://tcga-data.nci.nih.gov/tcga/) database.

## Abbreviations

HER2: Human epidermal growth factor receptor 2
BRCA: Breast cancer
TMB: Tumor mutational burden
ER: Estrogen receptor
PR: Progesterone receptor
TCGA: The Cancer Genome Atlas
WGCNA: Weighted gene co-expression network analysis
TOM: Topological overlap matrix
GO: Gene ontology
KEGG: Kyoto Encyclopedia of Genes and Genomes
DEGs: Differentially expressed genes
NMF: Negative matrix factorization
Tregs: T cells regulatory
ECM: Extracellular matrix
