# Supplementary material for: HER2 low expression breast cancer subtyping and their correlation with prognosis and immune landscape based on the histone modification related genes
Source: Sci Rep. 2023 Dec 8;13:21753. doi: 10.1038/s41598-023-49010-7 (PMC10709565; doi:10.1038/s41598-023-49010-7)
Supplement: Supplementary file 8 — Supplementary Figures. [file 41598_2023_49010_MOESM8_ESM.pdf]

# Her2

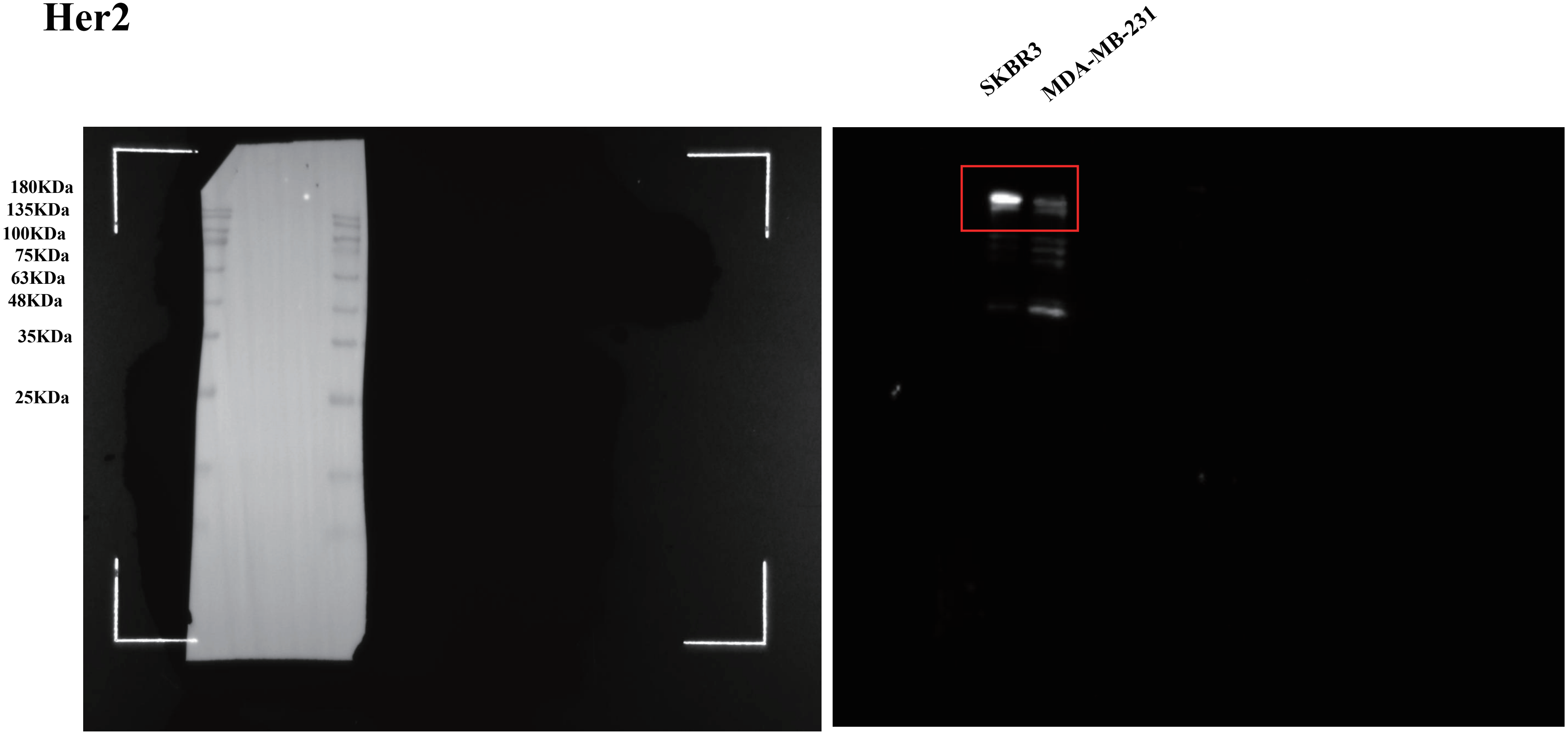
SKBR3
MDA-MB-231
180KDa
135KDa
100KDa
75KDa
63KDa
48KDa
35KDa
25KDa

NFKBIZ

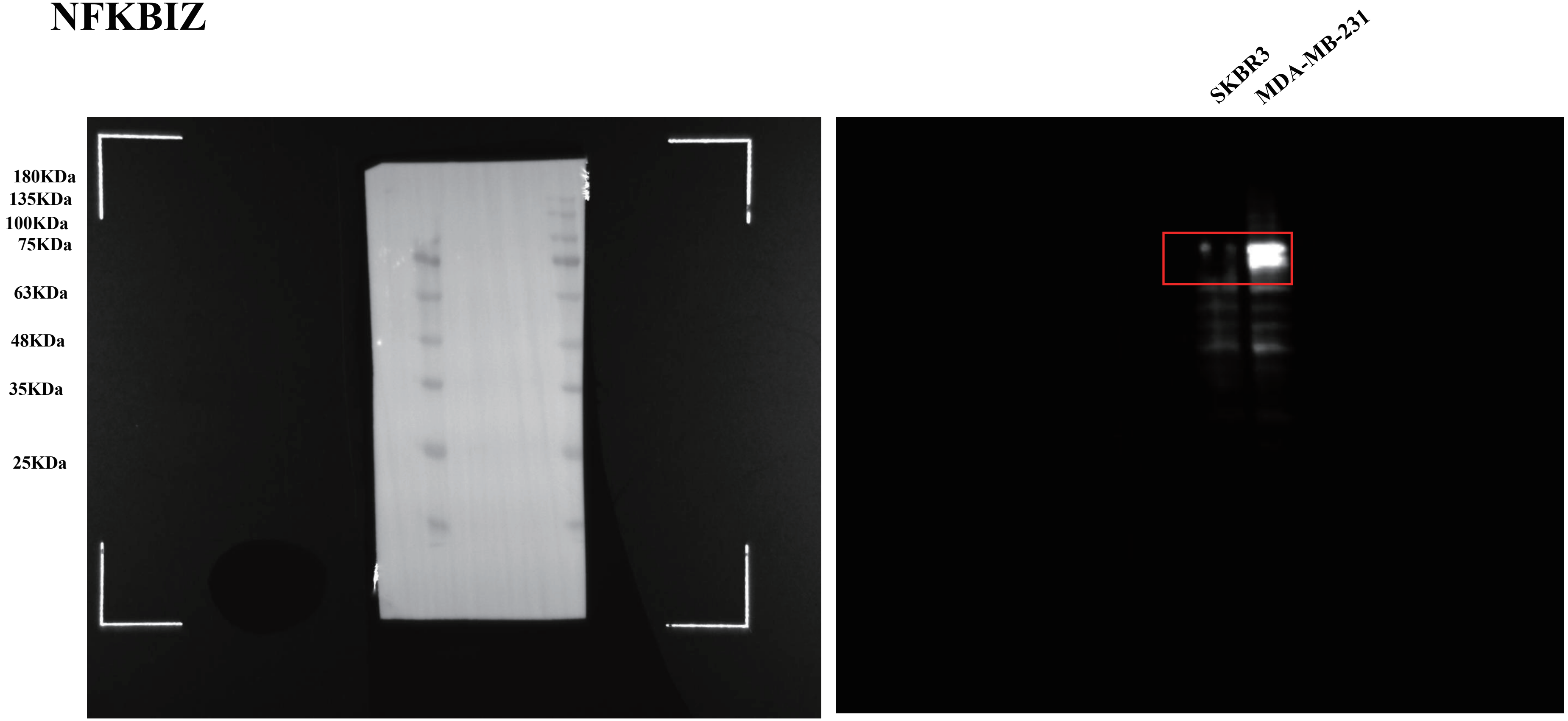
SKBR3
MDA-MB-231
180KDa
135KDa
100KDa
75KDa
63KDa
48KDa
35KDa
25KDa

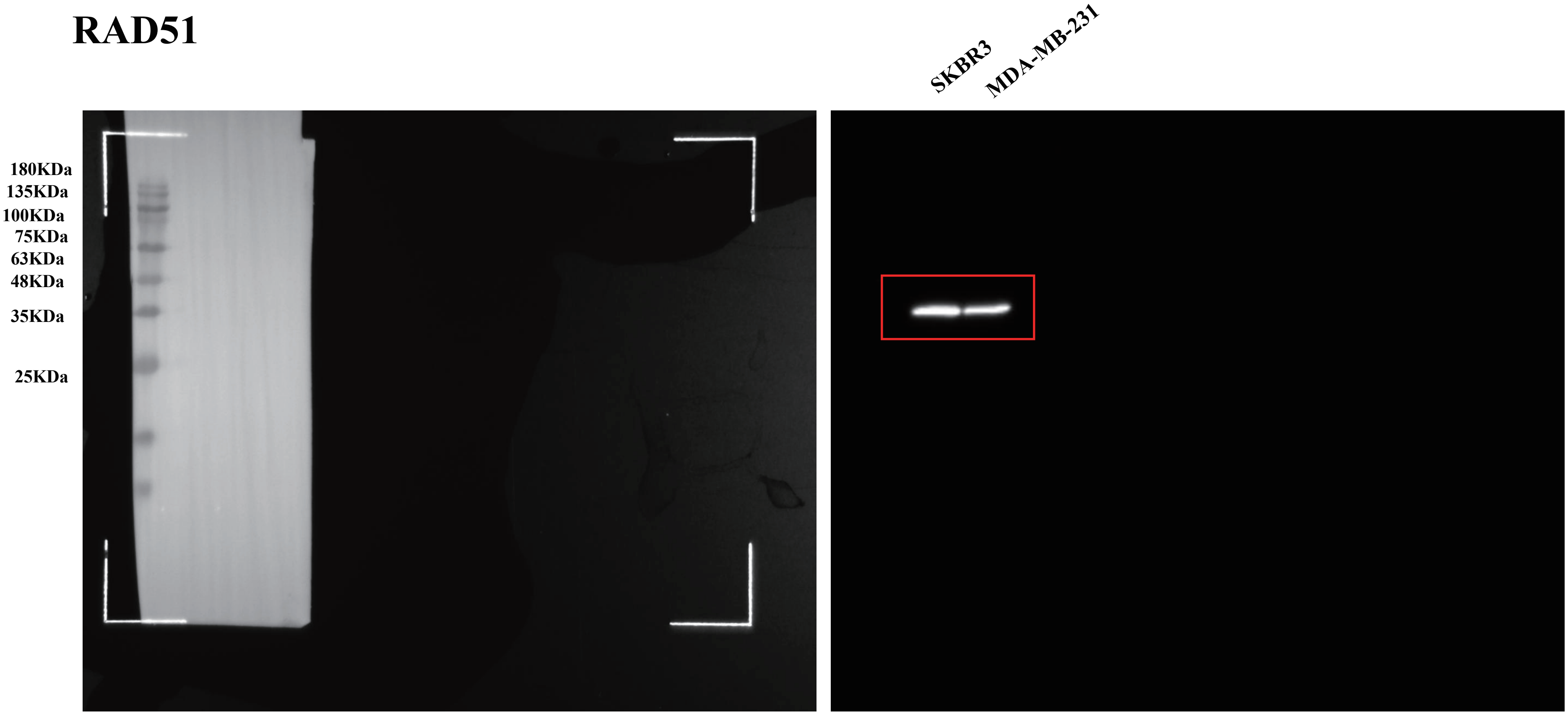
RAD51
180KDa
135KDa
100KDa
75KDa
63KDa
48KDa
35KDa
25KDa
SKBR3
MDA-MB-231

# GAPDH

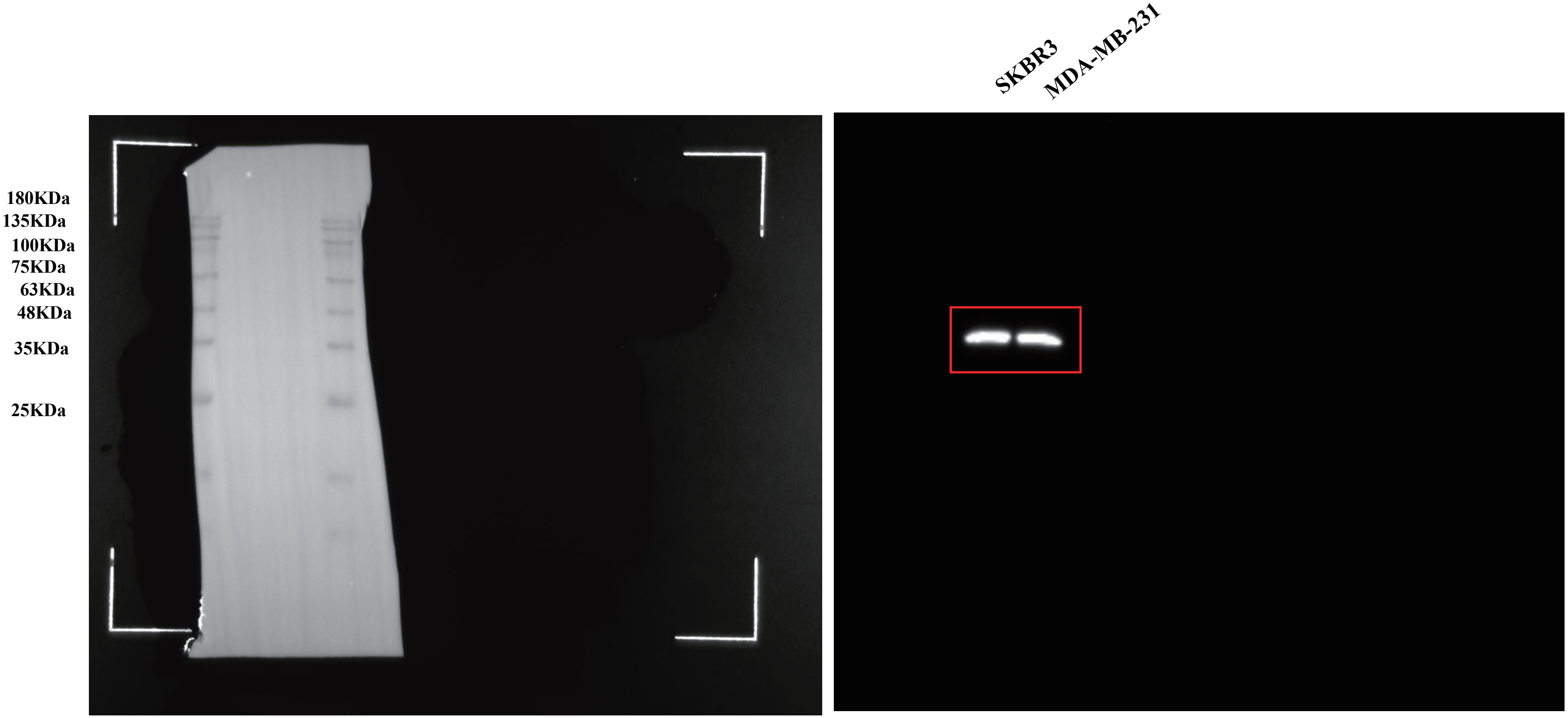
SKBR3
MDA-MB-231
180KDa
135KDa
100KDa
75KDa
63KDa
48KDa
35KDa
25KDa
